# The formation mechanism for printed silver-contacts for silicon solar cells

**DOI:** 10.1038/ncomms11143

**Published:** 2016-04-01

**Authors:** Jeremy D. Fields, Md. Imteyaz Ahmad, Vanessa L. Pool, Jiafan Yu, Douglas G. Van Campen, Philip A. Parilla, Michael F. Toney, Maikel F. A. M. van Hest

**Affiliations:** 1 National Renewable Energy Laboratory, 15013 Denver West Pkwy, Golden, Colorado 80401, USA; 2 SLAC National Accelerator Laboratory, 2575 Sand Hill Road, Menlo Park, California 94025, USA; 3 Department of Electrical Engineering, Stanford University, 350 Serra Mall, Stanford, California 94305, USA

## Abstract

Screen-printing provides an economically attractive means for making Ag electrical contacts to Si solar cells, but the use of Ag substantiates a significant manufacturing cost, and the glass frit used in the paste to enable contact formation contains Pb. To achieve optimal electrical performance and to develop pastes with alternative, abundant and non-toxic materials, a better understanding the contact formation process during firing is required. Here, we use *in situ* X-ray diffraction during firing to reveal the reaction sequence. The findings suggest that between 500 and 650 °C PbO in the frit etches the SiN_x_ antireflective-coating on the solar cell, exposing the Si surface. Then, above 650 °C, Ag^+^ dissolves into the molten glass frit – key for enabling deposition of metallic Ag on the emitter surface and precipitation of Ag nanocrystals within the glass. Ultimately, this work clarifies contact formation mechanisms and suggests approaches for development of inexpensive, nontoxic solar cell contacting pastes.

## Abstract (web summary)

The mechanism of contact formation during the firing of screen-printed contacts to Si solar cells remains elusive. Here, Fields *et al*. use in situ X-ray diffraction during firing to reveal the reaction sequence, thus suggesting approaches for development of inexpensive, nontoxic solar cell contacting pastes.

## Graphical abstract

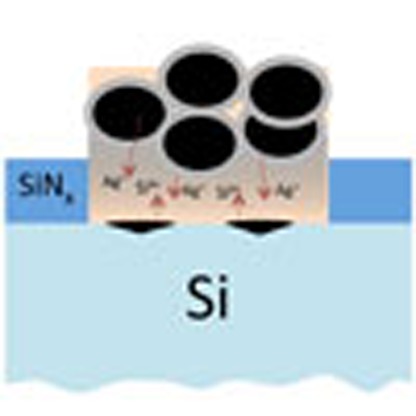

By far the most commercially viable option for photovoltaic energy generation, crystalline silicon (c-Si) continues to dominate the industry with over 90% market share. Optimally designed silver (Ag) front-contacts in the majority of c-Si solar cells utilize narrow grid lines (approximate width of 50 μm) to minimize shading loss and achieve high current, high fill factor, and hence, high photo-conversion efficiency. While screen-printing provides an economically attractive means for making these contacts, owing to suitability for high-volume manufacturing, the use of Ag adds significantly to the solar cell cost. Furthermore, despite more than three decades of use in photovoltaic manufacturing, the mechanism of contact formation during the firing of screen-printed contacts remains a subject of debate. Achieving optimal firing conditions to minimize contact resistance, and to develop new pastes with alternative materials (that is, earth-abundant and non-toxic), requires a detailed understanding of the contact formation process. *In situ* X-ray diffraction (XRD) data obtained during rapid-thermal processing (RTP) of Ag-paste materials reveal that there are multiple competing anti-reflective layer burn-through and Si oxidation reactions occurring with different temperature thresholds. The primary reactive species responsible for burn-through is lead-oxide (PbO), and these PbO driven redox reactions lead to liquid Pb formation and reversible Ag–Pb alloying during firing. Our *in situ* experiments also elucidate factors affecting dissolution of Ag into the frit and subsequent nanocrystal precipitation – key for achieving low contact resistance.

Typically, screen-printing contacts onto the n-type emitter of a p-type c-Si solar cell employs a paste consisting of Ag particles, organic binders and metal-oxide glass frit. The frit, usually a PbO-based borosilicate glass, promotes a series of reactions allowing electrical contact to the emitter after deposition of the silicon-nitride (SiN_x_) anti-reflective and passivation layer during contact firing. Although the frit has long been known to enable this process^1^, conflicting arguments bring into question the specific role of the frit to enable SiN_x_ burn-through and the mechanism of Ag transport to the c-Si surface. Hence, the actual electrical contact formation pathway remains scarcely understood – largely because previous investigations lacked *in situ* measurement capabilities with the temporal-resolution and operating temperature range necessary to observe these processes in real-time.

Reactions responsible for contact formation occur during rapid heating, with ramp-rates of 50–100 °C s^−1^, at temperatures of 500–800 °C. In general, the Ag-paste is well known to penetrate the SiN_x_ layer during firing by oxidation, and the resulting silicon-oxide (SiO_2_) reaction product becomes incorporated into the molten frit. However, the associated oxidation reaction remains in question, since this can occur in one of several ways. The two most frequently cited arguments suggest either that SiN_x_ is oxidized by PbO in the frit by^2^,^3^,^4^:


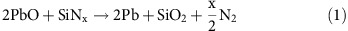


or, that Ag, which can dissolve in frit as silver oxide (Ag_2_O) up to approximately 5 wt.%(refs 5, 6), etches the SiN_x_ by^7^,^8^:


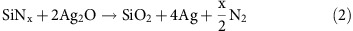


The second case is perhaps less obvious, since Ag_2_O itself readily decomposes under thermal activation, and so may not seem an obvious reactant at contact firing temperatures. However, Ag_2_O becomes more stable when bound in certain glasses, and therefore, technically both reactions (1) and (2) are thermodynamically feasible. *In situ* results presented in this work reveal the dominant SiN_x_ burn-through reaction.

After SiN_x_ opening, Ag contacts the Si emitter. Electrical conduction in fired contacts relies largely on tunneling through a layer of re-solidified glass frit, which forms between the c-Si surface and Ag bulk during firing. Distributed precipitation of Ag and Pb nanocrystals within the glass enables the tunneling^9^,^10^,^11^. Minimizing the glass intermediate layer thickness, inducing Ag sintering and promoting a controlled amount of Ag deposition on the c-Si surface (without spike-shunting the junction), are said to impart low contact resistance^12^. Here, certain additional details remain in question. For instance, the means by which Ag deposits onto the emitter is not obvious. According to some authors the deposition of Ag on the emitter requires the Ag to first oxidize by:


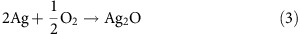


and then dissolve into the glass frit before a subsequent redox reaction between Ag_2_O and c-Si (refs 13, 14):


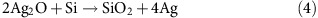


Not all agree with the Ag_2_O-based deposition model. Schubert (ref. 15) and Horteis^16^ argue that Pb formed in reaction 1 lowers the Ag–Si interaction temperature such that when the molten system phase separates upon cooling Ag deposits on the Si surface and within the glass layer. Sopori *et al*.^17^ agree with an Ag–Si alloy-assisted contact formation mechanism, and suggest involvement of other metal solvent species to further depress the melting temperature. In addition, the extent of Pb formation, argued to assist in Ag sintering, has been questioned. Thus, ambiguity persists regarding details of metal transport and precipitation, the extent of Ag–Pb alloying, the extent of Ag–Pb–Si alloying if any occurs at all, and therefore the keys for achieving low contact resistance.

Here, direct observation of SiN etching by PbO and Ag_2_O during different stages of firing using *in situ* XRD reveals the burn-through reaction and contact formation sequence. Studies on Ag, frit, c-Si and SiN model systems show a strong dependence of Pb formation, and hence Ag–Pb alloying, on oxygen (O_2_) partial pressure during firing. In addition, Ag dissolution into the glass frit, and hence the resulting abundance of Ag precipitate formation, shows a strong O_2_-dependence. This investigation finds the Ag redox reactions only occur with sufficient O_2_ at temperatures higher than 650 °C – negating a role of Ag in opening of the antireflective-coating at lower temperatures. Our findings also explain increased Pb formation observed when firing in a N_2_ atmosphere compared with air. Understanding the temperature thresholds for each of these competing mechanisms accurately clarifies the nature of screen-printed, fast-fired contacts.

## Results

### Powder model system

Model systems containing Ag nanocrystals, PbO-based frit, SiN_x_ and c-Si, in varying combinations, have been fired in an air or nitrogen (N_2_) atmosphere and characterized using an *in situ* XRD/RTP apparatus^18^. Use of powder model systems enabled characteristic XRD data acquisition with short collection intervals (<1 s), with high signal-to-noise achievable due to high interface area between reactants of interest within the sampled volume. This allowed detection of varying reaction species abundance with high sensitivity during rapid heating and cooling with temperature profiles analogous to solar cell contact firing conditions. This provides a unique opportunity to observe the reaction sequence in real time; such detailed analysis is impossible to perform on an actual Ag contact on a solar cell during firing in a belt-furnace. Results at the end of this section show that the powder mixture model systems fired by RTP in the present study behave analogous to fast-fired contacts made by screen-printing with conventional Ag pastes. Sample preparation and measurement procedures are explained further in the Methods section.

### Pb formation

**Figure 1:**
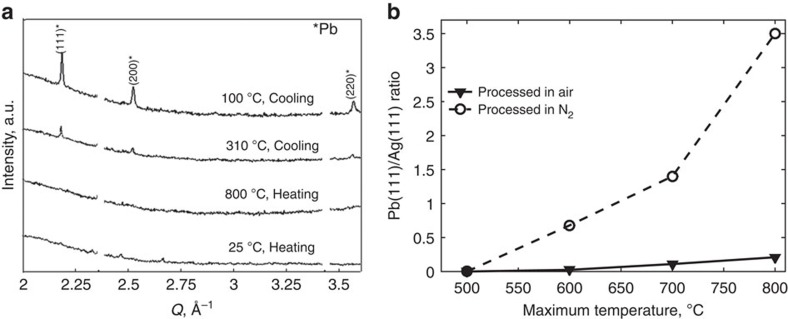
Lead formation dependence on firing temperature and atmosphere. (**a**) XRD patterns measured from frit/SiN_x_ during firing and cooling in air and (**b**) integrated Pb(111) diffraction intensity normalized by Ag(111) intensity observed upon firing Ag/frit/SiN_x_ mixtures to varying temperatures in air and N_2_.

XRD patterns measured during firing of a frit/SiN_x_ (1:1 molar ratio) mixture in air to 800 °C (Fig. 1a) demonstrate the *in situ* measurement capability. The frit and amorphous-SiN_x_ generate only weak, broad diffraction, in the initial patterns. However, sharp crystalline-Pb peaks emerge after firing, as the system cools below 327 °C (the melting temperature of Pb), which substantiates evidence of reaction 1. Results from analogous measurements on Ag/frit/SiN_x_ mixtures (Fig. 1b), fired to 500, 600, 700 and 800 °C, in air and N_2_, provide insights concerning the temperature threshold to initiate SiN_x_ etching, the accompanying Pb formation, and hence the extent of Ag–Pb alloying during contact firing.

As shown in Fig. 1b, which plots the ratio of Pb(111) integrated intensity to that of Ag(111), varying amounts of Pb form depending on both the maximum temperature and the firing atmosphere. Pb is not detected upon heating to 500 °C, whereas heating to 600 °C induces measurable Pb in both air and N_2_. Trend-lines show the amount of Pb increasing progressively with maximum firing temperature. Heating in N_2_ promotes Pb formation significantly compared with firing in air. Analogous behavior is observed in experiments with Ag/frit/c-Si, wherein PbO oxidizes c-Si by:


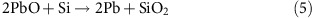


Enhanced Pb formation in N_2_ is explained by irreversible PbO reduction. In air, when Pb forms by reaction 1, it tends to react with O_2_, or Ag_2_O in the frit if present, and oxidize back to PbO by:


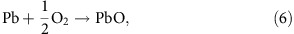


or:


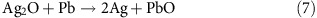


respectively. Thus, it seems regeneration of PbO by reactions 6 and 7 allows complete SiN_x_ etching despite a very small loading of PbO in the Ag paste (about 2 wt.%), as these reactions perpetuate the burn-through process.

### Burn-through reaction sequence revealed

**Figure 2:**
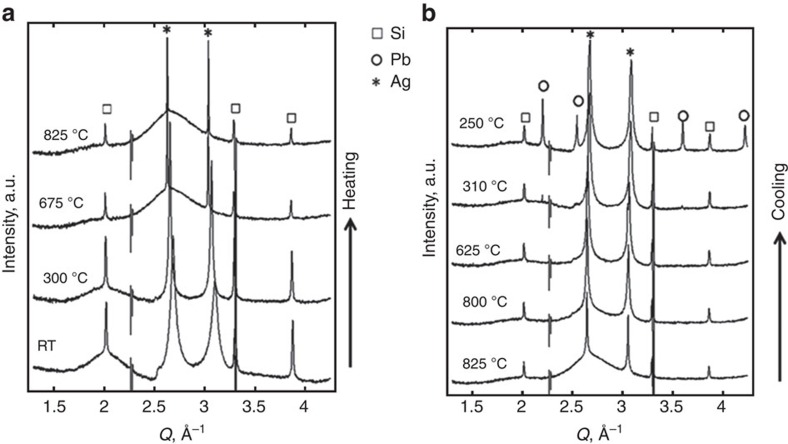
*In situ* diffraction measured from Ag/frit/SiN_x_/Si during firing. (**a**) XRD profiles obtained during heating to a max temperature of 825 °C at 50 °C s^-1^ and (**b**) cooling in air. Squares, circles, and stars indicate Si, Pb and Ag peaks, respectively. Note the changes in diffuse scattering from amorphous phases, near *Q*=2 Å^−1^ (attributed to glass frit) at low temperatures, and near *Q*=2.7 Å^−1^ (attributed to molten metal) at ∼675–825 °C.

Figure 2 shows diffraction patterns obtained from an Ag/frit/SiN_x_/Si mixture at different stages of firing. The Si diffraction decreases during heating between 550 and 750 °C, and does not recover upon cooling. This suggests oxidation of c-Si by Ag_2_O (reaction 4) and/or PbO (reaction 5). The Ag peaks initially narrow during temperature ramping as a consequence of Ag grain growth by sintering and ripening. Then on further heating to temperatures above 650 °C the Ag peak intensity diminishes, and a diffuse peak emerges near the Ag (111) peak (that is, at *Q*≅2.7 Å^−1^). The emerging diffuse peak is attributed to scattering from an amorphous phase, such as liquid metal (possibly Pb or Ag–Pb alloy), which is not present at room temperature and becomes most intense at the max temperature (825 °C) where the Ag peak intensity is a minimum. On cooling, the diffuse peak disappears while the Ag peaks gain intensity and broaden. Most likely, Ag peak broadening reflects contributions from newly formed Ag nanocrystals that precipitate within the glass during cooling. Pb peaks appear below 327 °C.

**Figure 3:**
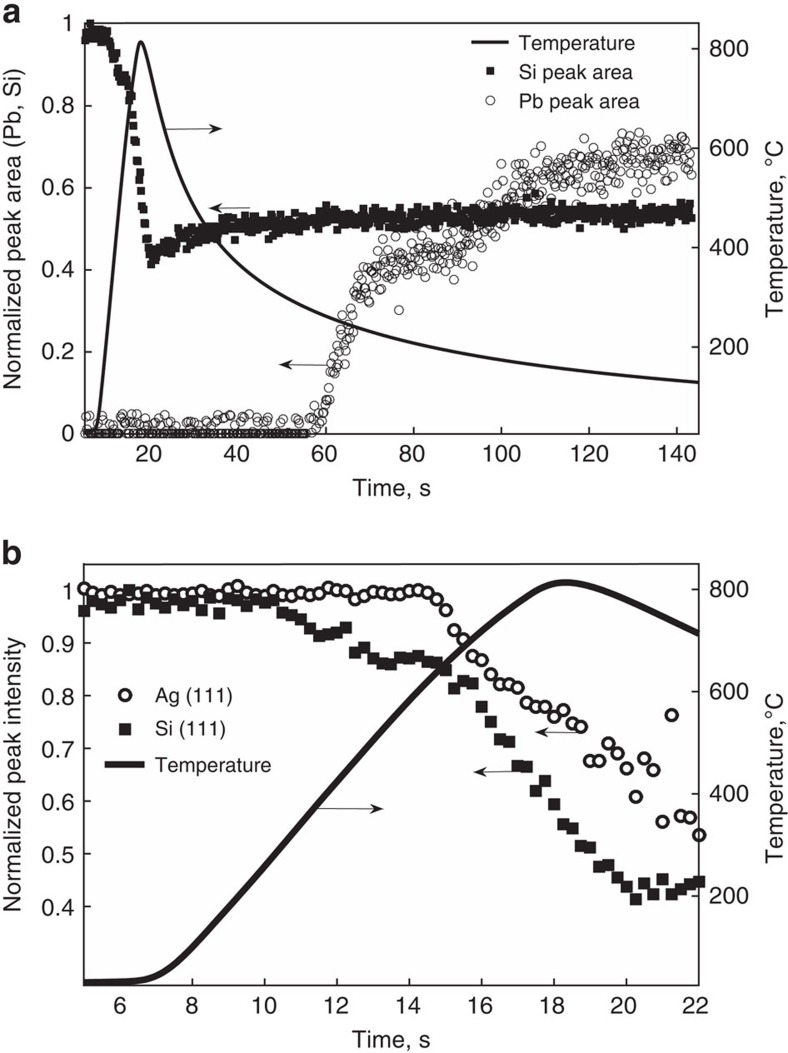
*In situ* diffraction from a model frit system during firing in air. (**a**) Temperature profile versus time (solid line) and corresponding variation in Si and Pb diffraction intensity during temperature ramping and cooling. (**b**) Si and Ag peak-area observed during firing, along with the temperature ramp profile (solid line).

Loss of Ag diffraction intensity during temperature ramping most likely follows from one of two possible mechanisms: Pb formed by redox reaction with SiN_x_ alloys and melts Ag, or Ag oxidizes and dissolves into the molten frit by reaction 3. In fact, the present study finds that both mechanisms occur, though with different onset temperatures. Examining the intensity trends versus time and temperature allows decoupling of the competing mechanisms. Figure 3 shows the corresponding temperature profile versus time along with Ag, Si and Pb integrated intensities. The Si diffraction intensity begins to decrease at about 10 s (500 °C). Then, the Ag peak intensity begins to diminish 5 s later, at about 14 s (650 °C). As the Ag signal begins to drop, the rate of Si signal loss accelerates markedly. Loss of Si signal results from Si oxidation by either PbO or Ag_2_O in the frit. Ag dissolution into the molten frit by reaction 3 must occur before etching by Ag_2_O (reaction 4). Since the Si signal decreases before the onset of Ag intensity loss, the low temperature oxidation (<650 °C) must be dominated by reaction with PbO in the frit (that is, by reaction 1). The observation of Pb diffraction upon cooling below 320 °C supports this interpretation, as this confirms formation of Pb.

Excellent agreement between the onset of Ag intensity decline and increased rate of Si oxidation at 15 s (650 °C) suggests a change in the dominant oxidation reaction during temperature ramping – most likely the onset of Si etching by Ag_2_O (reaction 4). Indeed, with Ag being the most noble metal in the system, Si oxidation by Ag_2_O, if present, should dominate given its thermodynamic favorability compared to reaction 1 (ref. 4).

### Involvement of Ag in SiN_x_ burn-through unlikely

**Figure 4:**
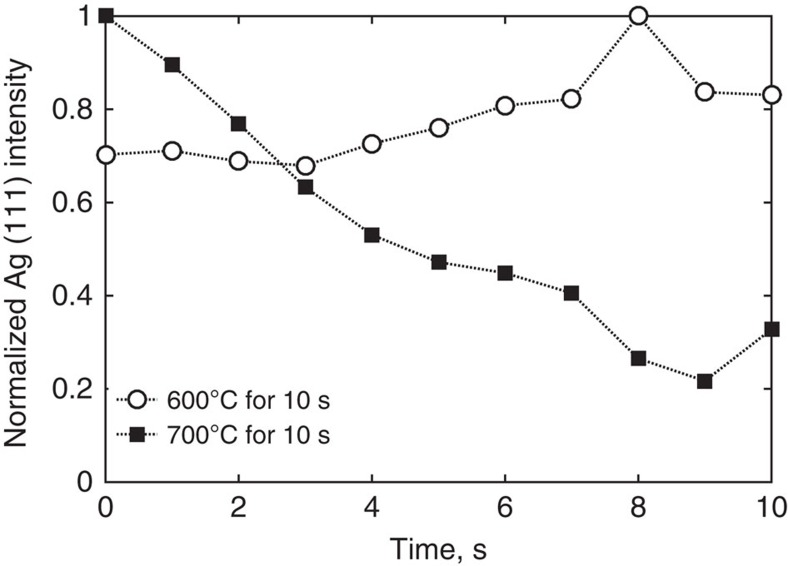
*In situ* measurement of Ag diffraction during isothermal heating. Ag(111) diffraction intensity as function of time during heating of Ag/frit (5 wt.% Ag) in air suggests Ag dissolution into the frit at 700 °C, but not at 600 °C.

Examining the nature of Ag dissolution into PbO-based frit under varying temperature conditions negates the possibility of Ag participation in SiN_x_ removal during the early stages of contact firing. To test this, Ag/frit mixtures (5 wt.% Ag) were isothermally annealed at 600 and 700 °C for 10 s. As shown in Fig. 4, annealing at 600 °C induces only a modest increase in Ag(111) intensity, probably due to grain growth. On the other hand, at 700 °C, the Ag intensity decreases and only 30% of the initial intensity remains after 10 s. The loss of Ag diffraction intensity implies about 70% Ag dissolution into the frit by reaction 3. Therefore, Ag dissolution into the frit must occur with a temperature threshold between 600 °C and 700 °C, and hence, oxidation of SiN_x_ or Si (observed onset temperature is approximately 500 °C) below this temperature range cannot involve Ag^+^ ions. The majority of SiN_x_ oxidation during temperature ramping must therefore be attributed to redox reactions with PbO.

### Silver transport

**Figure 5:**
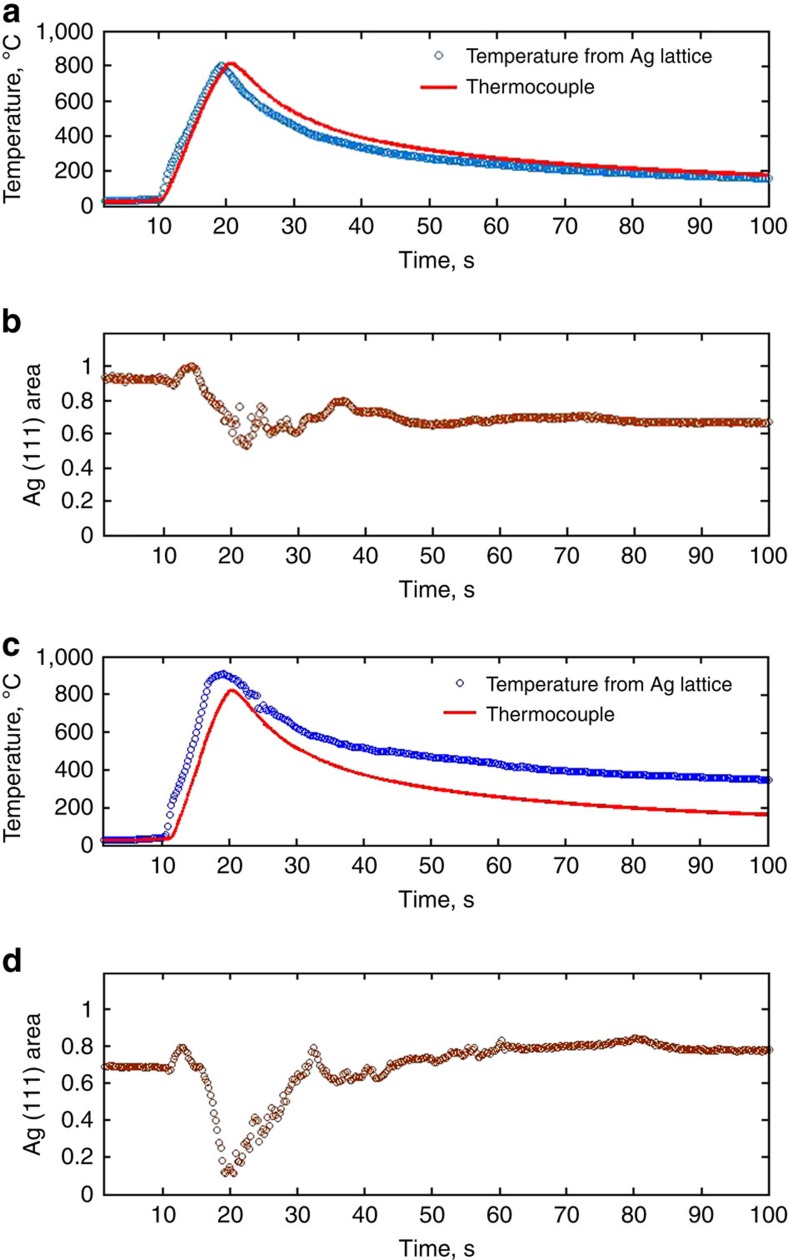
Temperature profiles and Ag(111) diffraction measured from Ag/frit/SiN_x_/Si during firing (100 °C s^−1^) and cooling in air and N_2_. (**a**,**c**) shows the temperature measured by thermocouple (red lines) versus calculated based on the Ag peak position (blue lines), and (**b**,**d**) shows the Ag diffraction intensity dependence on time. (**a**,**b**) corresponds to firing in air, while (**c**,**d**) is in N_2_.

Monitoring the Ag peak intensity during firing reveals the extent of Ag dissolution into the glass under varying conditions. Notably, the response of an Ag/frit/SiN_x_/Si mixture strongly depends on whether firing occurs in air or N_2_ (Fig. 5). During heating, the Ag peaks initially become sharper as a consequence of grain growth, and the intensity increases slightly. Then, above 650 °C (about 15 s), the Ag intensity diminishes continuously with increasing temperature up to the maximum temperature of 810 °C (about 20 s). In both cases, air and N_2_ atmosphere, the Ag peak intensity recovers during cooling, albeit to a different extent. In air, as applies in solar cell manufacturing, the Ag signal intensity decreases to approximately 50% at maximum temperature, and then recovers to about 75% of the initial value on cooling. On the other hand, when firing in N_2_, the Ag signal intensity drops to just 10% of the initial value at the maximum temperature, and then recovers completely upon cooling.

The different behavior in air versus N_2_ reflects the different forms of Ag during firing, respectively. According to the equilibrium Ag–Pb phase diagram, metallic Pb formed by redox reactions between PbO and SiN_x_ readily forms a liquid alloy with Ag in this temperature range. As was previously shown, more Pb forms in N_2_ due to irreversible reduction of PbO (Fig. 1b). Hence more Ag–Pb alloying occurs in N_2_, which is almost entirely reversible due to low miscibility of Ag and Pb solid phases at lower temperatures. On the other hand, Fig. 4 showed that Ag dissolves readily into the glass in the presence of O_2_ above 650 °C. Thus, in the case of firing in air, the dip in Ag signal reflects both Ag–Pb alloying and Ag dissolution, whereas only the former occurs in N_2_. In air, some of the Ag signal recovers as Ag nanocrystals precipitate within the glass upon cooling as a consequence of the temperature-dependent solubility. However, weak scattering from nanocrystals having a diameter below 2 nm, as are commonly seen in cross-sections of fired contacts^11^,^12^,^13^,^14^, and Ag remaining bound as Ag^+^ within the glass, are both consistent with lower Ag signal detected after firing in air.

**Figure 6:**
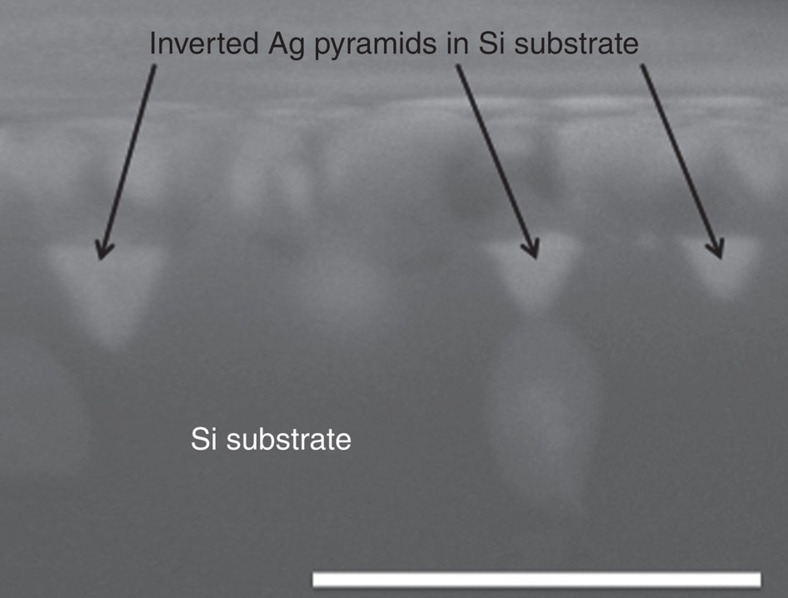
Cross section SEM showing Ag pyramids in a fired test sample. SEM image at × 100,000 magnification where the scale bar is 1 μm, shows a cross-section of an Ag/frit/SiN_x_ sample fired to 800 °C at 100 °C s^−1^ and reveals formation of inverted Ag pyramids penetrating the surface of the c-Si substrate.

Before considering implications of these results on screen-printed solar cell contacts, it is important to show that the model systems used in the present work behave representatively. Figure 6 shows a cross-section SEM of our model frit system with inverted Ag pyramids penetrating the surface of the c-Si substrate beneath the Ag/frit/SiN_x_ mixture after firing. The Ag pyramid formation observed here is analogous to that observed in numerous prior studies on fired Ag contacts^3^,^7^,^13^,^15^,^16^. Therefore, our frit, powder mixtures and firing conditions clearly mimic the screen-printed contact firing process. Interestingly, in this case, the mixture contained a larger proportion of frit (1:1 frit/Ag) compared with common Ag pastes used in solar cell contacts (typically lower than 0.1:1), which seems to have induced a larger characteristic Ag pyramid size compared with most other studies.

## Discussion

**Figure 7:**
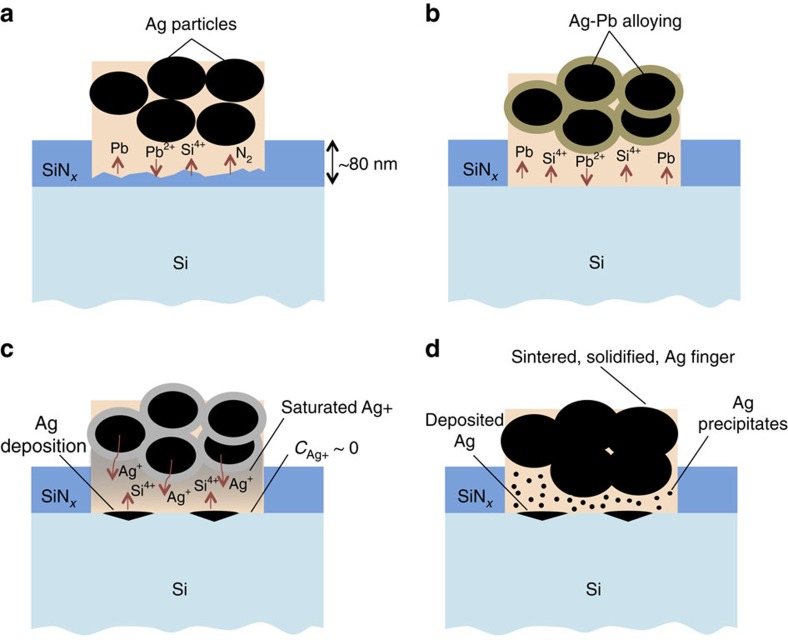
Illustrations showing stages of contact formation during firing. (**a**) SiN_x_ etching by PbO in the frit, (**b**) Ag–Pb alloying, (**c**) Ag^+^ transport through molten frit and deposition at the Si surface, and (**d**) resulting fired-contact morphology, with inclusion of small Ag precipitates within the glass intermediate layer.

A complete picture of the contact formation process, consistent with all previous findings and informed by the present *in situ* results, emerges as follows: during firing, above 500 °C, the frit melts and wets the Ag/SiN_x_ interface. Between 500 and 650 °C, PbO in the frit reacts with and penetrates the SiN_x_ anti-reflective layer by reaction 1, as shown in Fig. 7a. Pb formed during the burn-through process alloys with and assists in liquid-phase assisted sintering of Ag (Fig. 7b). Above 650 °C, Ag dissolves into the frit and diffuses towards the emitter surface. In the vicinity of the Si emitter surface, Ag ions are consumed by redox reaction 4, which oxidizes Si to SiO_2_ and deposits metallic Ag on the emitter surface (Fig. 7c). SiO_2_ formed by reaction 4 is incorporated into the molten frit. On cooling, the solubility of Ag in the melt decreases and nanocrystals precipitate from within the glass matrix. Kinetic constraints limit grain growth during fast cooling, resulting in a high density of distributed nanocrystals within the glass intermediate layer (Fig. 7d).

In summary, Ohmic contacts with low resistivity result when Ag crystals form within a thin glass intermediate layer and on the emitter surface; thus, in general, it would seem optimal contacts result with adequate heating to achieve complete SiN_x_ opening and abundant Ag precipitation within the glass. At the same time, over-firing must be avoided both to prevent excessive Ag deposition onto the emitter – Ag spikes – which can shunt the *pn*-junction in extreme cases, and to minimize SiO_2_ generation, which increases the thickness of the glass layer and diminishes carrier tunneling.

In the larger effort to replace costly Ag and toxic Pb in the solar cell manufacturing sector, the present study offers several insights. To replace Ag with an alternative, low-resistivity metal, assuming suitable work-function alignment for contacting Si, analogous use will require the metal have variable solubility in a frit system within a comparable temperature range (below 800 °C). The PbO-based frit may be replaced by a frit system containing another metal-oxide that alloys with the primary contact metal upon metal-oxide reduction and provides a low glass transition temperature. This is a necessary condition to achieve wetting and adhesion at the contact interface. The frit must contain a reactive species with a negative redox potential in relation to SiN_x_, and preferably to both SiN_x_ and Si. Ultimately, metal solubility and transport kinetics enabling nanocrystal precipitation within the glass upon cooling, which allows carrier tunneling (that is, ohmic contact formation), depends strongly on the frit composition. Thus, the metal and accompanying frit used in the paste must be developed in tandem.

## Methods

### Model system

Ag, frit, SiN_x_ and c-Si model systems in this study have been characterized as powder mixtures. Our model, controlled PbO-based frit was synthesized following ref. 15 by quenching a molten mixture of PbO (60 mol%), SiO_2_ (30 mol%) and B_2_O_3_ (10 mol%), and then milling to form a powder. Ag nanocrystals and SiN_x_ powder was purchased from Sigma-Aldrich, and c-Si nanocrystals were from Nanostructured and Amorphous Materials, Inc. Samples of varying composition were mixed to desired proportions in dry form, and then deposited onto 20 × 20 mm Si wafer substrates using isopropyl alcohol as a dispersing agent. Multiple samples were characterized for each test condition to ensure the reliability of the data.

### Measurement and heating

Our model systems were fired at heating rates around 100 °C s^−1^ and characterized by XRD in real-time using a synchrotron-mounted, RTP apparatus, equipped with a Pilatus, Dectris 300 K area detector, with 250 ms integration time (4 Hz sampling frequency). Specifics of the *in situ* XRD/RTP apparatus can be found elsewhere^18^. Briefly, the *in situ* RTP chamber was gold-coated aluminium enclosure with four high-power quartz-tungsten-halide lamps (750 W each). The chamber is sealed and plumbed to allow atmosphere control. Al coated Kapton windows were used to allow high X-ray transmission while containing heat during thermal processing. The RTP chamber body was water cooled by means of a chiller. Firing under N_2_ atmosphere was accomplished by purging the RTP chamber for 5 min prior to heating. *In situ* temperature measurements were obtained using a thermocouple, and cross-referenced based on calculations using the Ag diffraction signal (that is, the temperature dependence of the Ag lattice). This method provides a high level of accuracy in temperature measurement.

### Data analysis

Analysis of *in situ* XRD datasets was accomplished by fitting peaks with Pearson VII functions. The integrated area of the diffraction peak for a given phase corresponds directly with its abundance, which allowed extraction of phase-evolution trends versus time for the individual species of interest.

Cross-section SEM was performed using an FEI Nova NanoSEM, with a working-distance of 4.4 mm, accelerating potential of 10 kV and 1.3 mA operating current.

## Additional information

**How to cite this article:** Fields, J. D. *et al*. The formation mechanism for printed silver-contacts for silicon solar cells. *Nat. Commun.* 7:11143 doi: 10.1038/ncomms11143 (2016).

## References

[b1] Mertens R. . Critical processing parameter optimization for screen printed semicrystalline silicon solar cells. Proc. 17th IEEE PVSC 1347–1351 (1984).

[b2] Schubert G., Fischer B. & Fath P. Formation and nature of Ag thick film front contacts on crystalline silicon solar cells. Proc. PV Eur. Rome 343, 343–346 (2002).

[b3] Schubert G., Huster F. & Fath P. Physical understanding of printed thick-film front contacts of crystalline Si solar cells–review of existing models and recnet developments. Sol. Energy Mater. Sol. Cells 90, 3399–3406 (2006).

[b4] Horteis M., Gutberlet T., Reller A. & Glunz S. W. High-temperature contact formation on n-type silicon: basic reactions and contact model for seed-layer contacts. Adv. Funct. Mater. 20, 476–484 (2010).

[b5] Ueda S., Kumagai T. & Yamaguchi K. Thermodynamic study on the Ag–Pb–O system at 1273 K. Mater. Trans. 46, 1861–1864 (2005).

[b6] Ueda S., Kumagai T. & Yamaguchi K. Activity coefficient of AgO_0.5_ in the PbO–SiO_2_ melt at 1273 K. Mater. Trans. 48, 1458–1462 (2007).

[b7] Hong K. K. . Mechanism for the formation of Ag crystallites in the Ag thick-film contacts of crystalline Si solar cells. Sol. Energy Mater. Sol. Cells 93, 898–904 (2009).

[b8] Chung B. M. . Influence of oxygen on Ag ionization in molten lead borosilicate glass during screen-printed Ag contact formation for Si solar cells. Electrochim. Acta 106, 333–341 (2013).

[b9] Prudenziati M., Moro L., Morten B., Sirotti F. & Sardi L. Ag-based thick-film front metallization of silicon solar cells. Act. Pass. Elect. Comp. 13, 133–150 (1989).

[b10] Ballif C., Huljic D. M., Willeke G. & Hessler-Wyser A. Silver thick-film contacts on highly doped n-type silicon emitters: structural and electrical properties of the interface. Appl. Phys. Letts. 82, 1878–1880 (2003).

[b11] Hilali M. M. . Understanding the formation and temperature dependence of thick-film Ag contacts on high-sheet-resistance Si emitters for solar cells. J. ECS 152, 742–749 (2005).

[b12] Psych D., Mette A., Filipovic A. & Glunz S. W. Comprehensive analysis of advanced solar cell contacts consisting of printed fine-line seed layers thickened by silver plating. Prog. Photovoltaics 17, 101–114 (2009).

[b13] Hong K. K., Cho S. B., Huh J. Y., Park H. J. & Jeong J. W. Role of PbO-based glass frit in Ag thick-film contact formation for crystalline Si solar cells. Met. Mater. Int. 15, 307–312 (2009).

[b14] Cho S. B., Hong K. K., Huh J. Y., Park H. J. & Jeong J. W. Role of the ambient oxygen on the silver thick-film contact formation for crystalline silicon solar cells. Curr. Appl. Phys. 10, 222–225 (2010).

[b15] Schubert G. Thick Film Metallization of Crystalline Silicon Solar Cells PhD thesis University of Konstanz (2006).

[b16] Horteis M. Fine-Line Printed Contacts on Crystalline Silicon Solar Cells PhD thesis University of Konstanz (2009).

[b17] Sopori B. . Fundamental mechanisms in the fire-through contact metallization of Si solar cells: a review. in 17th Workshop on Crystalline Silicon Solar Cells and Modules: Materials and Processes; Workshop Proceedings No. NREL/BK-520-42056 (National Renewable Energy Laboratory (NREL), Golden, CO (2007).

[b18] Ahmad M. I. . Rapid thermal processing chamber for *in-situ* x-ray diffraction. Rev. Sci. Inst. 86, 130902-1–130902-7 (2015). 10.1063/1.4904848 25638092

